# Physically secure and fog-enabled lightweight authentication scheme for WBAN

**DOI:** 10.1038/s41598-025-16316-7

**Published:** 2025-08-21

**Authors:** Jegadeesan Subramani, Arun Sekar Rajasekaran, Arunkumar Balakrishnan, G. Anantha Rao

**Affiliations:** 1 Department of ECE, M.Kumarasamy College of Engineering, Karur, Tamilnadu India; 2 Department of ECE, SR University, Warangal, Telangana 506371 India; 3 Manipal Institute of Technology Bengaluru, Manipal Academy of Higher Education, Manipal, India; 4 Department of Electronics and Communication Engineering, Avanthi Institute of Engineering and Technology, Vizianagaram - 531 162, Cherukupally, Andhra Pradesh India

**Keywords:** Wireless body area network, Data privacy, System resilience, Fog computing, Session key management, Health care, Engineering

## Abstract

Wireless Body Area Networks (WBANs) are vital for healthcare, fitness monitoring, and remote patient care by means of combining sensors and wearable technologies for data collection and transmission. However, ensuring secure communication in WBANs remains a critical challenge and is generally insecure against the manipulation of data, breaches of privacy, and unauthorized access. Current authentication methods are vulnerable to security risks and have a significant computational burden. The above-said shortcomings are addressed by proposing a lightweight, physically secure, fog-enabled authentication scheme that guarantees data privacy and system resilience by integrating Physically Unclonable Functions ($\:PUFs$) and Fog Computing. This approach involves two phases: WBAN node registration and secure anonymous authentication. The proposed system incurs a reduction in computational overhead of 64.33% and communication overhead of 29.58% compared to existing protocols. Short-life session keys are used to achieve mutual authentication between WBAN sensors and monitoring devices. The proposed scheme is analyzed using BAN logic against attacks on impersonation, replay, and unauthorized access using BAN logic. Its practical effectiveness is confirmed via informal analysis, which shows that it is a scalable and efficient solution for practical WBAN environments.

## Introduction

The increased adoption of WBANs in healthcare, fitness, and emergency monitoring systems has been catalyzed by rapid progress in wearable technologies. Wearable or implantable sensor nodes constitute devices within a WBAN for monitoring vital physiological parameters (e.g. heart rate, blood glucose levels, body temperature, and motion)^1,2^. Such networks enable the use of applications ranging from remote health monitoring to personalized diagnostics and sports performance optimization with real-time data for medical professionals and improved patient care^3,4^.

However, increasing reliance on WBANs generates significant security and privacy challenges^5^. The sensitivity of medical data to attacks such as eavesdropping, data manipulation, and unauthorized access transmitted via wireless channels has been the focus of this research. However, the integration of WBANs into broader applications of IoT ecosystems intensifies these risks because of the commoditization of networked devices by malicious actors^6^. Moreover, WBAN sensor nodes are constrained by limited computational power, memory, and battery life, rendering the implementation of resource-intensive techniques such as RSA and ECC difficult^7,8^.

In response, fog computing has emerged as a promising paradigm for addressing these challenges by bringing data processing and decision-making closer to the network edge. Fog nodes reduce latency, supply real-time analytics, and increase security by isolating sensitive data before they enter centralized cloud servers^9^. In addition, adding $\:PUF$ to WBANs makes tamper resistance and device authentication easier. Using the device’s unique physical properties, $\:PUFs$ create unforgeable identifiers for WBAN nodes to prevent them from being cloned and forged^10^.

However, as the security challenges of WBANs, including unauthorized access, data breaches, and high energy consumption, have been induced by existing authentication mechanisms such as high computational overhead, vulnerability to replay attack, and low resource utilization, the need for an extremely lightweight and resource efficient authentication framework has evidently emerged. As a result, there was a motivation to integrate PUFs and Fog Computing to enhance security whilst reducing computational and communication efficiency, making the proposed solution well-suited for real-world WBAN deployments. Hence, we propose a novel fog-enabled PUF-based lightweight authentication framework for WBANs with limited resource constraints and security requirements. The contributions of this study are as follows.

1. PUF-Based Authentication: Using device-specific physical characteristics to resist tampering and device identification.
2. Fog-Enabled Architecture: Fog nodes perform real-time security management by decentralizing computational tasks.
3. Resource-efficient security: Reduces energy and computational overhead by employing lightweight cryptographic protocols compatible with low-power-constrained WBAN devices.

In contrast to prior WBAN security solutions, the proposed framework uniquely integrates fog computing with $\:PUF$-based authentication in a unified architecture to enhance resilience against both cyber and physical threats. The protocol introduces a stateless session key update mechanism using fresh nonces and real-time $\:PUF$ responses, ensuring perfect forward secrecy and immunity to desynchronization attacks, which are common challenges in traditional $\:PUF$ systems. Additionally, the system supports priority-based data routing, where normal condition data is directed to the cloud, and emergency data is transmitted immediately to medical personnel and caregivers. These features are further complemented by an offloading strategy, in which fog nodes handle security computations, allowing WBAN devices to preserve battery life and computing resources. These distinctive characteristics make the framework suitable for real-world, latency-sensitive healthcare applications with constrained edge devices.

The remainder of this paper is structured as follows: Section II contains an important literature review, Section III reports on previous work, and the proposed system model. Section IV presents an outline of the proposed framework and Section V presents a security analysis of this framework. Section VI compares the performance efficiency of the proposed scheme, and Section VII concludes the paper.

### Related works

WBAN is a wireless technology that can operate in a sensor network in highly sensitive environments, mainly in medical sections, where the treatment of sensitive patient data is essential. These networks are networks of wearables or implantable devices that continuously monitor physiological parameters and transmit this data to healthcare providers on time for decision-making. Although deployed in real-world scenarios, they face numerous security challenges such as impersonation attacks, replay attacks, session hijacking, and data tampering. Owing to the constrained computational resources, limited power supply, and dependence on wireless communication of WBANs, they are very vulnerable to malicious attacks. These security challenges have been documented extensively in previous studies. As an example, the authors in^11^ devise a lightweight authentication protocol appropriate for WBANs which strikes the right balance between security and energy efficiency. This protocol protects against lightweight impersonation and replay attacks with cryptographic inexpensiveness for low-power devices. In addition^12^, emphasized the growing potential of IoT-enabled vulnerabilities of WBANs and proposed the mitigation of sophisticated attacks through a multi-layered security approach. These studies revealed that the security mechanisms employed in WBANs need to be robust and energy efficient to ensure safe traffic of sensitive data in healthcare and other critical domains.

Although these problems have been addressed, many still need to be resolved. There are some key areas of further research in the form of ensuring that device pairing is secure, defending against physical tampering, and a schema similar to that of SNARC, which aims to balance security, energy efficiency, and scalability. Given their criticality in safety and security applications, these challenges are particularly acute in high-security applications, such as military operations or critical care unit applications.

WBAN security challenges have been successfully addressed by using fog computing as a novel paradigm. Fog computing extends scalability, responsiveness, and efficiency by decentralizing data processing to edge devices that are smaller than WBAN devices. This decentralized approach is desirable, especially in time-sensitive healthcare scenarios where the transmission or processing of data can cause severe consequences. Preprocessing, anomaly detection, and encryption can be performed at fog nodes by reducing the computational burden on WBAN devices and cloud servers. In^13^, the authors demonstrated that fog networking can enhance the security and performance of WBANs. In their study, they showed how fog nodes could mediate communications between WBAN devices and cloud servers in several ways, including layering on new means of authentication and encryption. With this approach, communication between resource-constrained WBAN nodes and cloud infrastructure mitigates risks, and real-time data processing is also increased.

However, fog-enabled WBAN frameworks have limitations. High-security applications, such as military operations and critical care settings, pose the danger of physical tampering of fog nodes and WBAN devices^13^. Fog nodes themselves may be targeted by cyberattacks and the entire network can be compromised. Eliminating these challenges necessitates the combination of solid physical security features with a strong authentication and encryption protocol. WBAN node security against physical and cyber threats requires the availability of physically unclonable functions (PUFs). Arrays of inherent manufacturing variant features, called PUFs, are exploited to produce unique, unclonable identifiers for use in device authentication and secure key generation. The effect of this hardware-based security mechanism is that it is not easily tampered with, as in an environment that has a lot of outside interference, which makes the results of this security mechanism more effective.

However, recent studies have demonstrated the security effectiveness of PUFs in WBAN. For instance, in^14^, the authors proposed a PUF-based authentication protocol that reduces the physical tampering risk and overall security. However, one aspect of the practical implementation of PUFs in WBANs is their noise resilience, scalability, and integration with other frameworks. PUF reliability is affected by environmental factors such as temperature and aging, and noise-resilient designs are required^15^. In addition, scaling PUF-based solutions for WSAN deployment is an open research problem. Finally, the lightweight nature of WBAN devices requires lightweight cryptographic protocols. Although robust, traditional cryptographic techniques tend to be too computationally intensive for devices with low processing power or short battery life. This gap is addressed using lightweight cryptographic protocols that guarantee the same type of security with minimal resource consumption.

In^17^, the authors proposed a hash-based authentication protocol for WBANs with reduced energy consumption, but it was still secure against impersonation and replay attacks. However, lightweight protocols already in existence^18^ tend to be largely missing from securing features such as multi-vector attack resistance or advanced persistent threat resistance. This limitation motivates adaptive cryptographic protocols that change the complexity according to the availability of resources and threat levels.

In addition to WBAN-specific security models, several authentication and key agreement protocols developed for related domains such as IoT, cloud computing, and wearable sensor networks are worth noting. For instance^19^, proposed a machine learning–resilient and low-latency authentication scheme for AI-driven patient monitoring, while^20^ developed a provably secure key management framework tailored for e-healthcare systems. The EAKE-WC protocol^21^ offers lightweight authenticated key exchange optimized for wearable computing, and^22^ presents a cloud-assisted secure and cost-effective solution for remote health monitoring. These works demonstrate cross-domain applicability, especially where low latency, energy efficiency, and robust authentication are critical. Integrating the design principles of these protocols with WBAN infrastructure may lead to enhanced security outcomes and promote the cross-employment of proven mechanisms across related platforms.

For example, the integration of these protocols with other security frameworks, such as fog computing and PUFs, will provide further security for WBAN. Although fog computing and PUF-based authentication have displayed great potential on individual fronts, their combinations have not been well explored^23^. Decentralized fog computing, in conjunction with hardware-based PUFs, forms a complementary addition that can be used to design robust frameworks for WBAN. Nevertheless, seamless interoperability, resource optimization, and scalability must be addressed to obtain effective integration.

The purpose of this study is to close this gap by proposing a hybrid fog-based and PUF-based lightweight authentication framework for WBANs. The balance between security, scalability, and resource efficiency is achieved through the proposed framework, which addresses the existing limitations in the approaches^23–26^. It offers a multilayer security mechanism to combat both cyber and physical attacks with little overhead by integrating fog computing and PUFs. Furthermore, the framework was optimized for energy consumption to conserve power in the resource-limited context of WBAN devices. This study contributes to the design and implementation of the hybrid framework, evaluation of the framework in real-world scenarios, and comparison of its performance against existing security solutions. These contributions are intended to promote the development of WBAN security and to serve as a basis for future work in the field of WBAN security.

### System model, preliminaries and attack model

#### System model

**Fig. 1:**
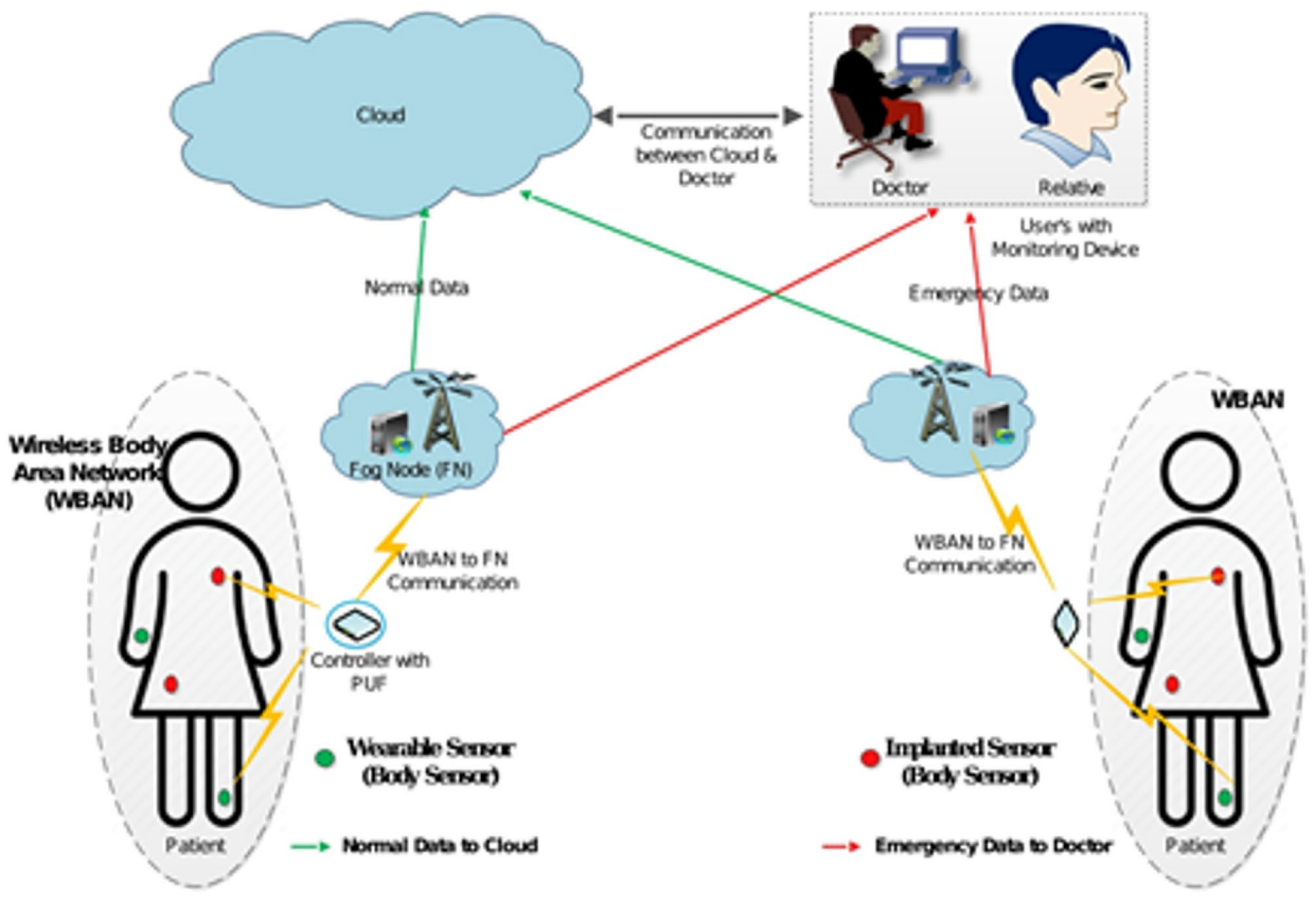
System model.

The adapted system model for the WBAN application is shown in Fig. 1 and consists of several core components such as Cloud Server $\:\left(CS\right)$, Fog Nodes $\:\left({FN}_{i}\right)$, WBAN Controller ($\:{WBAN}_{j}$) and Monitoring Device ($\:{MD}_{ij}$). The interaction of these elements results in the deployment of secure and efficient communication and monitoring in the WBAN environment.

##### Cloud server $\:\left(\varvec{C}\varvec{S}\right)$

In the WBAN framework, the $\:CS$ plays the role of a central trusted authority. The system uses services of the$\:CS$ that need to be used by both $\:{WBAN}_{j}$ and $\:{MD}_{ij}$, and in order to use services of the $\:CS$ these have to register with it and provide their credentials unique to them. Once registered, the $\:CS$ generates and distributes the initial security parameters necessary for mutual authentication, along with other parameters, is distributed to the client, to establish communication. The $\:CS$ is responsible for the overall security of the system including the detection and elimination of malicious activities, a primary repository for health aggregate data generated from the WBAN devices.

##### Fog node $\:\left({\varvec{F}\varvec{N}}_{\varvec{i}}\right)$

$\:{FN}_{i}$ is an intermediate between $\:{WBAN}_{j}$ and $\:{MD}_{ij}$. $\:{FN}_{i}$ is local to $\:{WBAN}_{j}$ and acts to increase system responsiveness and lower latency by performing localized computations and storage. In this framework, we call $\:{FN}_{i}$ a trusted entity, with enough computational power and memory to perform the authentication process and secure communication. In time-sensitive healthcare scenarios, its decentralized role improves the system’s efficiency and security.

To prevent $\:{FN}_{i}$ from becoming a single point of failure, the architecture supports redundancy using active-passive or load-balanced fog node deployment. Each fog node is independently preloaded with secure credentials, challenge-response pairs, and session parameters, ensuring continued operation even if another node fails. Additionally, lightweight anomaly detection mechanisms are embedded to detect suspicious behavior or compromise attempts, allowing the system to isolate affected nodes and reroute authentication tasks dynamically. This enhances system robustness against targeted cyberattacks.

##### WBAN controller ($\:{\varvec{W}\varvec{B}\varvec{A}\varvec{N}}_{\varvec{j}}$)

$\:{WBAN}_{j}$ is a body sensor interfaced with a central controller, and is the major node to be integrated within the WBAN system. Physiological data of patients, athletes, elderly persons, etc., are collected by body sensors, and health continues to be monitored by them. This data is consolidated by the $\:{WBAN}_{j}$ while also ensuring secure communication with the monitoring device. During the initial setup, each $\:{WBAN}_{j}$ list at the $\:CS$ to get the security parameters for authentication. Physically Unclonable Functions ($\:PUFs$) are used within the controller as a means to provide unique, device-specific identifiers, based on intrinsic hardware variations. These $\:PUFs$ offer robust hardware-based security, against unauthorized access or tampering attempts. The high level of tamper resistance is ensured by the fact that any attempt to manipulate the $\:PUFs$ makes them nonfunctional.

##### Monitoring device ($\:{\varvec{M}\varvec{D}}_{\varvec{i}\varvec{j}}$)

Doctors, medical professionals, relatives or any authorised individual uses the $\:{MD}_{ij}$ to monitor data collected by the $\:{WBAN}_{j}$. $\:{MD}_{ij}$ in the proposed scheme continuously links to the $\:{WBAN}_{j}$ which will provide real-time observation and analysis of the wearer’s health. $\:CS$ provide the basic secure parameters which are needed to utilize secure communication between $\:{MD}_{ij}$ and $\:{WBAN}_{j}$. $\:{MD}_{ij}$ talks to $\:{WBAN}_{j}$ via these secure channels to retrieve data in a way that it guarantees the integrity and confidentiality of the sensitive information. Additionally, $\:{MD}_{ij}$ also plays a critical important role in the transfer of device ownership. It performs handover authentication process in order to allow only allowed people to enter the system. For a confidential and secure transition of sensitive health data, this functionality is required. Monitoring Device provides a secure and efficient oversight mechanism within the framework of WBAN by fulfilling these responsibilities.

##### Scalability and device adaptability

The proposed protocol is scalable and supports dynamic changes in the number of WBAN devices. Each $\:{WBAN}_{j}$ independently initiates the authentication process using its unique identity and freshly generated session parameters. The protocol does not rely on global state synchronization, enabling seamless onboarding of new WBANs or removal of existing ones without affecting other active sessions. The session-specific use of nonces and timestamps ensures isolation and statelessness, which allows the system to accommodate fluctuating WBAN device counts efficiently.

#### Physically unclonable function ($\:\varvec{P}\varvec{U}\varvec{F}$)

The PUF is a microelectronic system that is designed to process the challenge $\:\left(C\right)$ and provide the response $\:\left(R\right)$. The $\:PUF$ produces an output denoted $\:R$ that is an outcome of the $\:PUF$ function, which takes the value $\:C$ as its input.

Consequently, the $\:PUF$ generates an output that differs from that of other systems with similar physical structures and inputs. Therefore, interference with the structural output of electronic devices is an effective way to protect their physical integrity, especially if they are located in remote areas. However, noise is a major flaw in the responsive action of the $\:PUF$. A reverse fuzzy extractor method has been successfully applied in eliminating noise from $\:PUF$ response with relatively low computation complexity^27^.

#### Attack model

The potential threats to the system under consideration were thoroughly investigated and meticulously analyzed for each specified attack scenario.

##### Physical intrusion attack

An adversary having physical access to $\:{WBAN}_{j}$ would attempt unauthorized access to tamper or steal the sensitive data, or devices. To overcome this, $\:{WBAN}_{j}$ employ tamper-evident security through PUFs. Safeguarding sensitive health data, any unauthorized attempt to access or manipulate the PUF makes the device non-functional.

##### Eavesdropping attack

In wireless communications between $\:{WBAN}_{j}$ and $\:{MD}_{ij}$, an interception by an adversary is an attempt to get hold of authentication credentials or sensitive health data. A secure, encrypted communication channel specified with parameters provided by the $\:CS$ is proposed using; Confidentiality and preventing unauthorized interception.

##### Man-in-the-middle attack

An adversary may impersonate devices, or change data during transmission in an attempt to intercept and modify communication between $\:{WBAN}_{j}$ and $\:{MD}_{ij}$. Besides PUF-based unique identifiers, the mutual authentication mechanism provides that only legitimate entities can participate in the communication. Additionally, no attempts to modify or falsify data are allowed, providing integrity taken with the data.

##### Resource-exhaustion attack

An adversary might be able to increase performance over $\:{WBAN}_{j}$ computational and communication resources to degrees in which performance is degraded or services are denied. The system continues operating even with attacks, through the development of lightweight cryptographic protocols that guarantee functionality and the optimization of resource allocation mechanisms to minimize its energy consumption.

##### Dolev-Yao attack model

In the Dolev-Yao model an adversary is allowed to eavesdrop, intercept, modify, fabricate, and retransmit messages. Specifically designed to confront these advanced threats through robust encryption, secure key-exchange protocols, and PUF-based authentication mechanisms, the proposed scheme attempts to counter these issues. These measures enable the WBAN system to remain confidential, well-known, and authentic, even in a severely insecure environment.

### Proposed scheme

The proposed approach consists of three main stages: system initialization, user registration, and subsequent authentication.

#### System initialization

##### Master key generation

To secure communication and authentication amongst different entities in the system, the Cloud Server ($\:CS$) generates master secrets. The aforementioned master secrets are $\:{s}_{CS}$ (the master key for Cloud Server), $\:{s}_{{FN}_{i}}$ (shared key for Fog Nodes) and $\:{s}_{{WBAN}_{j}}$ (the key associated with WBAN Controllers only). These keys comprise the basis of security in the system, from which they create secure channels and trusted interactions among the components.

##### $\:\varvec{P}\varvec{U}\varvec{F}$ challenge-response pair ($\:\varvec{C}\varvec{R}\varvec{P}$) registration

For CRP registration, each device $\:\left({WBAN}_{j},\:{MD}_{ij}\right)$ has to perform a one-time secure registration with $\:CS$ in a physically secure and trusted setup (e.g., certified hospital environment or factory provisioning). Each of these challenges ($\:C$) are then applied to the $\:PUF$ resulting in unique responses $\:{R}_{PUF}=PUF\left(C\right)$ for challenges in this process. Then the $\:\left(C,\:{R}_{PUF}\right)$ challenge-response pairs are securely transmitted to the $\:CS$.

To prevent eavesdropping or tampering during this transfer, CRPs are encrypted using an ephemeral session key derived as $\:{K}_{Session}^{Initial}=H({R}_{PUF}\parallel\:{s}_{CS})$, or transferred via a trusted, offline method. The $\:CS$ stores these CRPs for future authentication.

##### Preloading parameters

The $\:CS$ preloads essential security parameters into the devices, including CRP subsets and device-specific keys. These parameters are protected during preloading using either physical security controls or cryptographic encryption, ensuring confidentiality and integrity before deployment.

##### Secure parameter distribution

$\:CS$ distribute initialization keys and corresponding $\:CRPs$ with Fog Nodes ($\:{FN}_{i}$) in a secure manner. Specifically, $\:CS$ shares $\:\left\{{C}_{{FN}_{i}},\:\:{R}_{{PUF}_{{FN}_{i}}},\:\:{s}_{{FN}_{i}}\right\}\to\:{FN}_{i}$ either during a trusted offline provisioning phase or via a secure, authenticated encryption channel. This ensures confidentiality, integrity, and protection against CRP exposure during the distribution process, enabling decentralized and tamper-resistant authentication.

#### System entity registration

The Entity Registration phase ensures that $\:{WBAN}_{j}$​ and $\:{MD}_{ij}$​ securely register with $\:CS$ to establish their identity and receive initial parameters for authentication.

### WBAN controller ($\:{\varvec{W}\varvec{B}\varvec{A}\varvec{N}}_{\varvec{j}}$) registration

#### Step 1:

$\:{WBAN}_{j}$generates its unique identifier $\:{UID}_{{WBAN}_{j}}$ and secret $\:{m}_{{WBAN}_{j}}$​.

#### Step 2:

Compute the $\:PUF$ Response as $\:{R}_{{PUF}_{{WBAN}_{j}}}=PUF\left({C}_{{WBAN}_{j}}\right)$, and calculate the Hash value as $\:{M}_{{WBAN}_{j}}=H\left({UID}_{{WBAN}_{j}}\parallel\:{m}_{{WBAN}_{j}}\right)$.

#### Step 3:

Send the Registration Request $\:\left\{{UID}_{{WBAN}_{j}},\:\:{M}_{{WBAN}_{j}},\:\:{R}_{{PUF}_{{WBAN}_{j}}},\:\:{C}_{{WBAN}_{j}}\right\}$ to $\:CS$.

**Step 4**: $\:CS$ verifies $\:{M}_{{WBAN}_{j}}$​​ and registers $\:{WBAN}_{j}$​. Assigns the following parameters: $\:{\alpha\:}_{{WBAN}_{j}}=H\left({UID}_{{WBAN}_{j}}\parallel\:{s}_{{WBAN}_{j}}\right)$, $\:{K}_{Session}^{Initial}=H\left({R}_{{PUF}_{{WBAN}_{j}}}\parallel\:{s}_{CS}\right)$.

### Monitoring device ($\:{\varvec{M}\varvec{D}}_{\varvec{i}\varvec{j}}$​) registration

#### Step 1:

$\:{MD}_{ij}$​ generates Identity and Secret key as follows, Unique identifier $\:\left({UID}_{{MD}_{ij}}\right)$, Password $\:\left({PW}_{{MD}_{ij}}\right)$​ and Random nonce $\:\left({N}_{{MD}_{ij}}\right)$.

#### Step 2:

Compute the Temporary Identifiers as $\:{TID}_{{MD}_{ij}}=H\left({UID}_{{MD}_{ij}}\parallel\:{N}_{{MD}_{ij}}\right)$, $\:{TPW}_{{MD}_{ij}}=H\left({TID}_{{MD}_{ij}}\parallel\:{PW}_{{MD}_{ij}}\right)$.

#### Step 3:

Send the Registration Request $\:\left\{{TID}_{{MD}_{ij}},\:\:{UID}_{{MD}_{ij}},\:\:{C}_{{MD}_{ij}}\right\}$ to $\:CS$.

**Step 4**: $\:CS$ verifies $\:{TID}_{{MD}_{ij}}$​​ and registers $\:{MD}_{ij}$​. Assigns the following parameters: $\:{\alpha\:}_{{MD}_{ij}}=H\left({TID}_{{MD}_{ij}}\parallel\:{s}_{{FN}_{i}}\right)$, $\:{\delta\:}_{{MD}_{ij}}=PUF\left({C}_{{MD}_{ij}}\right)\oplus{TPW}_{{MD}_{ij}}$, $\:{K}_{Session}^{Initial}=H\left({R}_{{PUF}_{{MD}_{ij}}}\parallel\:{s}_{CS}\right)$.

### Authentication scheme

The authentication scheme ensures session-key uniqueness, forward secrecy, and secure communication while preventing session-key reuse. It incorporates provisions for dynamic parameters, session identifiers, and device-specific keys to ensure secure and efficient operation. The notation used in the proposed method is listed in Table 1.

**Table 1:** Notations and description.

Notation	Description
$\:{UID}_{{MD}_{ij}}$	Unique ID of Monitoring Device $\:{MD}_{ij}$
$\:{UID}_{{WBAN}_{j}}$	Unique ID of WBAN Controller $\:{WBAN}_{j}$
$\:{N}_{{MD}_{ij}}$ and $\:{N}_{{WBAN}_{j}}$	Dynamic nonces generated by $\:{MD}_{ij}$​ and $\:{WBAN}_{j}$​, respectively
$\:{TS}_{i}$	Timestamp for freshness verification
$\:SID$	Session Identifier
$\:{K}_{Session}$	Current session key
$\:{K}_{Session}^{Prev}$	Previous session key
$\:{R}_{{MD}_{ij}}$ and $\:{R}_{{WBAN}_{j}}$	PUF-based responses from $\:{MD}_{ij}$ and $\:{WBAN}_{j}$
$\:MAC$	Message Authentication Code
$\:H\left(x\right)$	Cryptographic hash function

**Step 1**: Request Initialization by $\:{MD}_{ij}$​ by​ generating a fresh nonce $\:{N}_{{MD}_{ij}}$​ and a timestamp $\:{TS}_{i}$. Next, it computes the following, $\:{R}_{{MD}_{ij}}=PUF\left({C}_{{WBAN}_{j}}\right)\oplus{H}\left({TID}_{{MD}_{ij}}\parallel\:\:{N}_{{MD}_{ij}}\right)$, $\:SID=H\left({N}_{{MD}_{ij}}\parallel\:{N}_{{WBAN}_{j}}\parallel\:{TS}_{i}\right).$ Finally, it Sends the following request to $\:{WBAN}_{j}$​ via the Fog Node $\:\left({FN}_{i}\right)$: $\:{M}_{request}=\left\{{UID}_{{MD}_{ij}},\:\:{R}_{{MD}_{ij}},\:\:SID,{\:TS}_{i}\right\}\:$to $\:{WBAN}_{j}$.

**Step 2**: $\:{WBAN}_{j}$​ receives $\:{M}_{request}$​ and validates the $\:{\:TS}_{i}$by ensuring $\:\left|{TS}_{j}-{TS}_{i}\right|\le\:\triangle{T}$ to prevent replay attacks and also it verifies the $\:SID$ by ensuring $\:SID=H\left({N}_{{MD}_{ij}}\parallel\:{N}_{{WBAN}_{j}}\parallel\:{TS}_{i}\right)$. Next, it computes, $\:{R}_{{MD}_{ij}}^{{\prime\:}}=PUF\left({C}_{{WBAN}_{j}}\right)\oplus{H}\left({TID}_{{MD}_{ij}}\parallel\:\:{N}_{{MD}_{ij}}\right)$. If $\:{R}_{{MD}_{ij}}^{{\prime\:}}={R}_{{MD}_{ij}}$​, then $\:{MD}_{ij}$​ is authenticated. Further, it generates a fresh nonce $\:{N}_{{WBAN}_{j}}$​, response $\:{R}_{{WBAN}_{j}}=PUF\left({C}_{{WBAN}_{j}}\right)\oplus{H}({UID}_{{WBAN}_{j}}\parallel\:\:{N}_{{WBAN}_{j}})$ and sends the following response to $\:{MD}_{ij}$​: $\:{M}_{response}=\left\{{UID}_{{WBAN}_{j}},\:{R}_{{WBAN}_{j}},\:SID,\:{N}_{{WBAN}_{j}}\right\}$

#### Step 3

$\:{MD}_{ij}$​ verifies $\:{R}_{{WBAN}_{j}}^{{\prime\:}}=PUF\left({C}_{{WBAN}_{j}}\right)\oplus{H}({UID}_{{WBAN}_{j}}\parallel\:\:{N}_{{WBAN}_{j}})$. If $\:{R}_{{WBAN}_{j}}^{{\prime\:}}={R}_{{WBAN}_{j}}$​, then $\:{WBAN}_{j}$​ is authenticated. Next, both $\:{MD}_{ij}$​ and $\:{WBAN}_{j}$ compute the session key as $\:{K}_{Session}=H\left({R}_{{MD}_{ij}}\parallel\:{R}_{{WBAN}_{j}}\parallel\:{K}_{Session}^{Prev}\parallel\:SID\right)$, ensuring it incorporates fresh parameters and the previous session key for uniqueness.

**Step 4**: During communication, the request, response, and verification of messages are structured as follows: Each message includes $\:SID$ and a Message Authentication Code, $\:MAC=H({K}_{Session}\parallel\:M)$, where $\:M$ is the message content. The transmitted request is: $\:{M}_{transmitted}=\left\{M,\:MAC,\:SID\right\}$. The response message includes an updated $\:{SID}^{{\prime\:}}$ and its $\:MAC$: $\:MAC=H({K}_{Session}\parallel\:{M}_{response})$, The transmitted response is: $\:{R}_{transmitted}=\left\{{M}_{response},\:{MAC}^{{\prime\:}},\:{SID}^{{\prime\:}}\right\}$. For verification, the receiver ensures: $\:SID=H\left({N}_{{MD}_{ij}}\parallel\:{N}_{{WBAN}_{j}}\parallel\:{TS}_{i}\right)$, and $\:MAC=H({K}_{Session}\parallel\:M)$.

**Step 5**: Session key update mechanisms are as follows: Both $\:{MD}_{ij}$ and $\:{WBAN}_{j}$​ generate new nonces $\:{N}_{{MD}_{ij}}^{{\prime\:}}$ and $\:{N}_{{WBAN}_{j}}^{{\prime\:}}$ and compute the New Session Identifier as $\:{SID}^{{\prime\:}}=H\left({N}_{{MD}_{ij}}^{{\prime\:}}\parallel\:{N}_{{WBAN}_{j}}^{{\prime\:}}\parallel\:{TS}_{i}^{{\prime\:}}\right)$. Next, it updates the Session Key as $\:{K}_{Session}^{New}=H\left({R}_{{MD}_{ij}}^{{\prime\:}}\parallel\:{R}_{{WBAN}_{j}}^{{\prime\:}}\parallel\:{SID}^{{\prime\:}}\parallel\:{K}_{Session}^{Prev}\right)$. Finally, both devices do the continuity verification by ensuring the following: $\:{K}_{Session}^{New}=H\left({R}_{{MD}_{ij}}^{{\prime\:}}\parallel\:{R}_{{WBAN}_{j}}^{{\prime\:}}\parallel\:{SID}^{{\prime\:}}\parallel\:{K}_{Session}^{Prev}\right)$.

### Security analysis

This section evaluates the security effectiveness of the proposed approach by conducting both formal and informal analyses to address the various security risks.

#### Formal security analysis

The suggested approach is evaluated by Burrows, Abadi, and Needham ($\:BAN$) logic to assess its security properties.

The postulates of the $\:BAN$ logic is given as follows.

Message-meaning rule ($\:{R}_{1}$) : $\:\frac{P|\equiv\:P\underleftrightarrow{K}Q,P\lhd{\left\{X\right\}}_{K}}{P|\equiv\:Q|\sim\:X}$

$$\:\text{N}\text{o}\text{n}\text{c}\text{e}-\text{v}\text{e}\text{r}\text{i}\text{f}\text{i}\text{c}\text{a}\text{t}\text{i}\text{o}\text{n}\:\text{r}\text{u}\text{l}\text{e}\:\left({R}_{2}\right)\::\:\frac{P|\equiv\:\#\left(X\right),P|\equiv\:Q|\sim\:(X)}{P|\equiv\:Q|\equiv\:X}$$

Jurisdiction rule $$\:\left({R}_{3}\right)$$ : $$\:\frac{P\left|\equiv\:Q\right|\Rightarrow\:X,P|\equiv\:Q|\equiv\:X}{P|\equiv\:X}$$

Freshness rule $$\:\left({R}_{4}\right)$$ : $$\:\frac{P|\equiv\:\#(X)}{P|\equiv\:\#(X,Y)}$$

Belief rule $$\:\left({R}_{6}\right)$$ : $$\:\frac{P|\equiv\:Q|\equiv\:(X,Y)}{P|\equiv\:Q|\equiv\:\left(X\right)}$$

The following are the preliminary security assumptions for the suggested approach.

Let $\:{WBAN}_{j}$​ be the WBAN Controller, and $\:{MD}_{ij}$​ the Monitoring Device.

##### Assumptions

• $\:{WBAN}_{j}$​ and $\:{MD}_{ij}$​ share $\:{K}_{Session}$​, derived securely through the protocol.

- Nonces $\:{N}_{{WBAN}_{j}}$ and ​$\:{N}_{{MD}_{ij}}$ are fresh and random.
- $\:SID$ is fresh and unique to each session.
- $\:{WBAN}_{j}$​ and $\:{MD}_{ij}$ believe that each other has jurisdiction over the session key.

**Logical beliefs**:

- $\:{WBAN}_{j}|\equiv\:\#{N}_{{WBAN}_{j}}$​: $\:{WBAN}_{j}$ believes that the nonce $\:{N}_{{WBAN}_{j}}$​ is fresh.
- $\:{MD}_{ij}|\equiv\:\#{N}_{{MD}_{ij}}$: $\:{MD}_{ij}$​ believes that the nonce $\:{N}_{{MD}_{ij}}$ is fresh.
- $\:{MD}_{ij}|\equiv\:\#SID$: $\:{MD}_{ij}$​ believes that the session identifier $\:SID$ is fresh.
- $\:{WBAN}_{j}|\equiv\:\#SID$: $\:{WBAN}_{j}$​ believes that the session identifier $\:SID$ is fresh.
- $\:{WBAN}_{j}|\equiv\:{WBAN}_{j}\underleftrightarrow{{K}_{Session}}{MD}_{ij}$: $\:{WBAN}_{j}$​ believes that it securely shares the session key $\:{K}_{Session}$​ with $\:{MD}_{ij}$​.
- $\:{MD}_{ij}|\equiv\:{MD}_{ij}\underleftrightarrow{{K}_{Session}}{WBAN}_{j}$: $\:{MD}_{ij}$​ believes that it securely shares the session key $\:{K}_{Session}$​ with $\:{WBAN}_{j}$​.

To show how the suggested approach is secure enough, you would need to comply with at least these specific requirements,

$$\:{G}_{1}$$: **Mutual authentication**.

- $\:{WBAN}_{j}|\equiv\:{MD}_{ij}|\equiv\:{WBAN}_{j}\underleftrightarrow{{K}_{Session}}{MD}_{ij}$: $\:{WBAN}_{j}$​ believes that $\:{MD}_{ij}$​ shares the session key $\:{K}_{Session}$​.
- $\:{MD}_{ij}|\equiv\:{WBAN}_{j}|\equiv\:{WBAN}_{j}\underleftrightarrow{{K}_{Session}}{MD}_{ij}$ : $\:{MD}_{ij}$​ believes that $\:{WBAN}_{j}$​ shares the session key $\:{K}_{Session}$​.

$$\:{G}_{2}$$: **Key secrecy**.

- $\:{WBAN}_{j}|\equiv\:{WBAN}_{j}\underleftrightarrow{{K}_{Session}}{MD}_{ij}$: $\:{WBAN}_{j}$​ believes that the session key $\:{K}_{Session}$ is securely shared with $\:{MD}_{ij}$.
- $\:{MD}_{ij}|\equiv\:{MD}_{ij}\underleftrightarrow{{K}_{Session}}{WBAN}_{j}$ : $\:{MD}_{ij}$​ believes that the session key $\:{K}_{Session}$​ is securely shared with $\:{WBAN}_{j}$​.

$$\:{G}_{3}$$: **Freshness of** $$\:{\varvec{K}}_{\varvec{S}\varvec{e}\varvec{s}\varvec{s}\varvec{i}\varvec{o}\varvec{n}}$$.

- $\:{WBAN}_{j}|\equiv\:\#\left({K}_{Session}\right)$: $\:{WBAN}_{j}$​ believes that the session key $\:{K}_{Session}$​ is fresh.
- $\:{MD}_{ij}|\equiv\:\#\left({K}_{Session}\right)$: $\:{MD}_{ij}$ believes that the session key $\:{K}_{Session}$​ is fresh.

The following steps are used to obtain the anonymous authentication (among $\:{WBAN}_{j}$ and $\:{MD}_{ij}$ using the above criteria assisted with assumptions).

##### Protocol idealized messages

Let $\:{WBAN}_{j}$ be the WBAN Controller, and $\:{MD}_{ij}$ the Monitoring Device.

**The idealized protocol steps are expressed as follows**:

M1: $\:{MD}_{ij}$ to $\:{WBAN}_{j}$: $\:\left\{{UID}_{{MD}_{ij}},\:\:{R}_{{MD}_{ij}},\:\:SID,{\:TS}_{i}\right\}$, where $\:{R}_{{MD}_{ij}}=PUF\left({C}_{{WBAN}_{j}}\right)\oplus{H}\left({TID}_{{MD}_{ij}}\parallel\:\:{N}_{{MD}_{ij}}\right)$, and $\:SID=H\left({N}_{{MD}_{ij}}\parallel\:{N}_{{WBAN}_{j}}\parallel\:{TS}_{i}\right)$.

M2: $$\:{WBAN}_{j}$$ **to** $$\:{MD}_{ij}$$: $$\:\left\{{UID}_{{WBAN}_{j}},\:{R}_{{WBAN}_{j}},\:SID,\:{N}_{{WBAN}_{j}}\right\}$$, where $$\:{R}_{{WBAN}_{j}}=PUF\left({C}_{{WBAN}_{j}}\right)\oplus{H}({UID}_{{WBAN}_{j}}\parallel\:\:{N}_{{WBAN}_{j}})$$.

**Step 1**: $\:{\varvec{M}\varvec{D}}_{\varvec{i}\varvec{j}}$**​ sends M1 to** $\:{\varvec{W}\varvec{B}\varvec{A}\varvec{N}}_{\varvec{j}}$:

1. $$\:{WBAN}_{j}$$ computes $$\:{R}_{{MD}_{ij}}^{{\prime\:}}=PUF\left({C}_{{WBAN}_{j}}\right)\oplus{H}\left({TID}_{{MD}_{ij}}\parallel\:\:{N}_{{MD}_{ij}}\right)$$.
2. If $\:{R}_{{MD}_{ij}}^{{\prime\:}}={R}_{{MD}_{ij}}\to\:{WBAN}_{j}\lhd{R}_{{MD}_{ij}}$ and infers: $\:{WBAN}_{j}|\equiv\:{MD}_{ij}|\sim{R}_{{MD}_{ij}}$ (message meaning rule R1).
3. Since $\:{WBAN}_{j}|\equiv\:\#{N}_{{MD}_{ij}}\to\:$ By Nonce Verification Rule (R2): $\:{WBAN}_{j}|\equiv\:{MD}_{ij}|\equiv\:{R}_{{MD}_{ij}}$.
4. $\:SID$ check: $\:SID=H\left({N}_{{MD}_{ij}}\parallel\:{N}_{{WBAN}_{j}}\parallel\:{TS}_{i}\right)$, Since $\:SID$ binds both nonces and timestamp: $\:{WBAN}_{j}|\equiv\:\#SID$.

**Step 2**: $\:{\varvec{W}\varvec{B}\varvec{A}\varvec{N}}_{\varvec{j}}$ **sends M2 to** $\:{\varvec{M}\varvec{D}}_{\varvec{i}\varvec{j}}$.

1. $$\:{MD}_{ij}$$ receives $$\:\left\{{UID}_{{WBAN}_{j}},\:{R}_{{WBAN}_{j}},\:SID,\:{N}_{{WBAN}_{j}}\right\}$$, computes $$\:{R}_{{WBAN}_{j}}^{{\prime\:}}=PUF\left({C}_{{WBAN}_{j}}\right)\oplus{H}({UID}_{{WBAN}_{j}}\parallel\:\:{N}_{{WBAN}_{j}})$$.
2. ​If $$\:{R}_{{WBAN}_{j}}^{{\prime\:}}={R}_{{WBAN}_{j}}\to\:{MD}_{ij}|\equiv\:{WBAN}_{j}|\sim{R}_{{WBAN}_{j}}$$.
3. $\:{MD}_{ij}|\equiv\:\#{N}_{{WBAN}_{j}}\to\:$ By Nonce Verification Rule (R2): $\:{MD}_{ij}|\equiv\:{WBAN}_{j}|\equiv\:{R}_{{WBAN}_{j}}$.
4. Both agree on $\:SID$ → shared session context.

**Step 3: Session key agreement**.

1. Both $\:{MD}_{ij}$ and $\:{WBAN}_{j}$​ computes $\:{K}_{Session}=H\left({R}_{{MD}_{ij}}\parallel\:{R}_{{WBAN}_{j}}\parallel\:{K}_{Session}^{Prev}\parallel\:SID\right)$.
2. Since both $\:{MD}_{ij}$ and $\:{WBAN}_{j}$​ believe that $\:{R}_{{MD}_{ij}}$, $\:{R}_{{WBAN}_{j}}$ values and $\:SID$ are fresh:

$$\bullet\:\:\:\:\:\:{MD}_{ij}|\equiv\:\#\left({K}_{Session}\right)$$

$$\bullet\:\:\:\:\:\:{WBAN}_{j}|\equiv\:\#\left({K}_{Session}\right)$$

By Jurisdiction Rule (R3):

- $$\:{MD}_{ij}|\equiv\:{WBAN}_{j}\Rightarrow\:{K}_{Session}$$, and $$\:{MD}_{ij}|\equiv\:{WBAN}_{j}|\equiv\:{K}_{Session}\to\:{MD}_{ij}|\equiv\:{K}_{Session}$$.
- Similarly, $$\:{WBAN}_{j}|\equiv\:{MD}_{ij}|\equiv\:{K}_{Session}$$.

$\:{WBAN}_{j}$​ and $\:{MD}_{ij}$​ achieve mutual authentication. Both entities believe that $\:{K}_{Session}$​ is securely shared and authenticated. Further, $\:{K}_{Session}$ is securely derived and shared exclusively between $\:{WBAN}_{j}$​ and $\:{MD}_{ij}$​​. The use of fresh nonces $\:\left\{{N}_{{WBAN}_{j}},\:\:{N}_{{MD}_{ij}}\right\}$ and unique session identifiers $\:\left(SID\right)$ ensures that $\:{K}_{Session}$ remains fresh and unique for every session.

#### Informal security analysis

##### Mutual authentication

Certain values of cryptographic verification are used in the flow of the proposed protocol to accomplish the $\:{WBAN}_{j}$​ and $\:{MD}_{ij}$​ authentication. Each of the entities checks the identity of the counterpart through challenge-response values generated by the $\:PUFs$. By using dynamic nonces $\:{N}_{{WBAN}_{j}},\:\:{N}_{{MD}_{ij}}$, and unique $\:SID$, the protocol ensures that only authorized entities can engage in safe communications, making it easy to achieve mutual authentication.

##### Replay attack resistance

Replay attacks are prevented by using time-bound session-specific tokens $\:\left({TS}_{i}\right)$ and distal nonce $\:\left({N}_{{WBAN}_{j}},\:\:{N}_{{MD}_{ij}}\right)$ for every communication cycle. To ensure freshness, the receiver validates the timestamp by checking whether $\:\left|{TS}_{j}-{TS}_{i}\right|\le\:\varDelta\:T$, where $\:{TS}_{j}$ is the local time and $\:\varDelta\:T$ is a predefined threshold that accommodates network latency, processing delays, and possible clock drift in wireless environments. In case an adversary $\:\left(\varLambda\:\right)$ tries to resume an eavesdropped session, the timestamps or the nonces is checked and found invalid and thus, the session is denied. This dynamic approach eliminates any possibility of the system being undermined by old or duplicate messages.

##### Impersonation attack resistance

The proposed scheme helps to avoid impersonation attacks because the responses and cryptographic keys are generated from the $\:PUF$ of a device. An adversary $\:\left(\varLambda\:\right)$ who wants to impersonate either $\:{WBAN}_{j}$​ or $\:{MD}_{ij}$ would require open access to secret keys $\:\left({s}_{{WBAN}_{j}},\:\:{s}_{{MD}_{ij}}\right)$ and unique responses $\:\left({R}_{{WBAN}_{j}},\:{R}_{{MD}_{ij}}\right)$ which are safely stored out of reach of the adversary. But, the inclusion of the cryptographic parameters for instance hashed challenge-response pair makes it possible to thwart any impersonation.

##### Session key secrecy

Session keys ($\:{K}_{Session}$), are also dynamic and are computed based on other parameters such as nonces $\:\left({N}_{{WBAN}_{j}},\:\:{N}_{{MD}_{ij}}\right)$, $\:PUF$response and session ID $\:\left(SID\right)$ for each communication session. In an additional level of security, all subsequent session keys derived from the current master key will not be affected in any way should an opponent get hold of an earlier session key. It ensures forward secrecy so that at the end of each session no means can be traced to subsequent sessions.

##### User anonymity

$\:{TID}_{{MD}_{ij}}$ preserving user anonymity through the dynamically updated pseudonymous identifiers. These identifiers are refreshed between each session avoiding an adversary $\:\left(\varLambda\:\right)$ from linking communication sessions to specific users. Hence, in the proposed scheme, the secret information remains hidden, even when transmitted over a public channel.

##### Forward secrecy

The suggested scheme achieves forward secrecy, in which a new session key $\:{K}_{Session}$ is established for each session, and is dependent on fresh inputs such as nonces $\:\left({N}_{{WBAN}_{j}},\:\:{N}_{{MD}_{ij}}\right)$ and PUF responses $\:\left({R}_{{WBAN}_{j}},\:{R}_{{MD}_{ij}}\right)$. If long-term keys (resp. credentials) are ever compromised past session keys cannot be reconstructed and retroactive attacks are shielded against communication past while keys have remained valid. It guarantees a very high level of data confidentiality that does not change over time.

##### Resistance to physical attacks

Inherent resistance to physical attacks is achieved by using $\:PUFs$. If the adversary $\:\left(\varLambda\:\right)$ can tamper the device to extract cryptographic parameters, then the $\:PUF$ would be nonfunctional and the stored data useless. Also, $\:PUFs$ possess additional tamper detection mechanisms that notify the system if there is a probable breakage.

##### Insider attack resistance

Even though an insider registers as a legitimate user, they cannot recover the session keys of other users as $\:PUF$ responses and cryptographic credentials are unique to a device. Sensitivity of parameters, which can be sensitive inputs like $\:{\alpha\:}_{{MD}_{ij}}=H\left({TID}_{{MD}_{ij}}\parallel\:{s}_{{FN}_{i}}\right)$, depends on inputs that must be securely distributed and are inaccessible to unauthorized entities.

##### Resistance to temporary secret key leakage

The protocol resists temporary secret key leakage because the computation of session keys involves independent unique parameters like nonces $\:\left({N}_{{WBAN}_{j}},\:\:{N}_{{MD}_{ij}}\right)$, $\:PUF$responses $\:\left({R}_{{WBAN}_{j}},\:{R}_{{MD}_{ij}}\right)$, and cryptographic keys. Although $\:\varLambda\:$ gets at least some data (such as a partial nonce), without complete knowledge of all necessary data, an adversary is not able to deduce the session key.

##### Resistance to cloning attacks

Device-specific $\:PUFs$ guarantee that no two devices have the same challenge-response behaviour. Cloning of devices is prevented by this, as duplication of a $\:PUF$ is computationally infeasible. In the domain of $\:PUFs$, any attempt to mimic a genuine device would inevitably fail because $\:PUF$ responses are unique and unpredictable.

##### Resistance to desynchronization attacks

The proposed authentication scheme resists desynchronization attacks by design, as it avoids storing or updating any state-dependent variables across sessions. Each authentication instance independently uses fresh nonces $\:{N}_{{MD}_{ij}}$ and $\:{N}_{{WBAN}_{j}}$ along with a timestamp $\:{TS}_{i}$ to derive the session identifier $\:SID=H\left({N}_{{MD}_{ij}}\parallel\:{N}_{{WBAN}_{j}}\parallel\:{TS}_{i}\right)$. The $\:PUF$-based responses $\:{R}_{{MD}_{ij}}=PUF\left({C}_{{WBAN}_{j}}\right)\oplus{H}\left({TID}_{{MD}_{ij}}\parallel\:\:{N}_{{MD}_{ij}}\right)$ and $\:{R}_{{WBAN}_{j}}=PUF\left({C}_{{WBAN}_{j}}\right)\oplus{H}({UID}_{{WBAN}_{j}}\parallel\:\:{N}_{{WBAN}_{j}})$ are computed afresh for each session and not reused. The session key $\:{K}_{Session}=H\left({R}_{{MD}_{ij}}\parallel\:{R}_{{WBAN}_{j}}\parallel\:{K}_{Session}^{Prev}\parallel\:SID\right)$ is also derived dynamically without requiring synchronization of counters or helper data. As all parameters are freshly generated and not dependent on previous session success, failed or dropped sessions do not cause state mismatch, thus ensuring desynchronization resistance.

### Performance analysis

In this section, we analyze the performance efficiency of the proposed approach by examining parameters such as the computational overhead, communication overhead, and security attributes.

#### Computational overhead

To analyze the computational overhead of the proposed scheme, the time consumed by the important cryptographic operations such as the hash function $\:\left({T}_{H}\right)$, $\:PUF$ $\:\left({T}_{PUF}\right)$, reverse fuzzy extractor $\:\left({T}_{F}\right)$, scalar multiplication $\:\left({T}_{M}\right)$ and symmetric cryptography $\:\left({T}_{S}\right)$ of the suggested protocols are considered in this section. The proposed approach is evaluated using Ubuntu 14.04 VMware with an Intel Core i5-8265U processor and an 8-GB RAM system. The simulation work was carried out using the JCE library Pbc-05.14 and the computational overhead of different cryptographic operations, such as $\:{T}_{H}$, $\:{T}_{PUF}$, $\:{T}_{F}$, $\:{T}_{M}$, and $\:{T}_{S}$ are calculated as 0.011 $\:ms$ $\:\left(millisecond\right)$, 0.12 $\:ms$, 2.53 $\:ms$, 2.6 $\:ms$, and 0.041 $\:ms$ respectively. Table 2 lists the computational overhead of various methods, and it ensures that the suggested approach consumes only 3.024 $\:ms$ to establish a session key, whereas other related existing approaches^23–26^, consume 8.47 ms, 8.04 ms, 5.77 ms, and 15.79 ms as a communication overhead.

**Table 2:** Computational overhead of various schemes.

Methods	$\:{\varvec{W}\varvec{B}\varvec{A}\varvec{N}}_{\varvec{j}}$	$\:{\varvec{F}\varvec{N}}_{\varvec{i}}$	$\:{\varvec{M}\varvec{D}}_{\varvec{i}\varvec{j}}$	Total
^24^	$\:{2T}_{S}+{8T}_{H}+{T}_{F}$	$\:{5T}_{S}+{11T}_{H}+{T}_{F}$	$\:{2T}_{S}+{6T}_{H}+{T}_{F}+{2T}_{PUF}$	$\:{9T}_{S}+{25T}_{H}+{3T}_{F}+{2T}_{PUF}=8.474ms$
^25^	$\:{14T}_{H}+{2T}_{F}+{T}_{PUF}$	$\:{8T}_{H}$	$\:{8T}_{H}+{T}_{F}$	$\:{30T}_{H}+{3T}_{F}+{T}_{PUF}=8.04ms$
^26^	$\:{T}_{S}+{10T}_{H}+{T}_{F}$	$\:{4T}_{S}+{9T}_{H}$	$\:{3T}_{S}+{5T}_{H}+{T}_{F}+{T}_{PUF}$	$\:{8T}_{S}+{24T}_{H}+{2T}_{F}+{T}_{PUF}=5.772ms$
^27^	$\:{3T}_{M}+{6T}_{H}$	$\:{T}_{M}+{6T}_{H}$	$\:{2T}_{M}+{6T}_{H}$	$\:{6T}_{M}+{18T}_{H}=15.798ms$
Suggested scheme	$\:{15T}_{H}$	$\:{12T}_{H}$	$\:{7T}_{H}+{T}_{F}+{T}_{PUF}$	$\:{34T}_{H}+{T}_{F}+{T}_{PUF}=3.024ms$

It is important to note that the performance evaluation was conducted on a system with Intel Core i5-8265U processor and 8 GB RAM running Ubuntu 14.04, selected to represent a constrained computing environment typical of edge or wearable WBAN devices. While this configuration helps simulate real-world resource limitations, we acknowledge that results may vary on modern edge computing platforms with advanced processors (e.g., ARM Cortex-A76, Intel i7/i9, or Raspberry Pi 5-level SoCs). Performance profiling on such contemporary hardware platforms will be considered in our future work to generalize the findings across broader deployment scenarios.

**Fig. 2:**
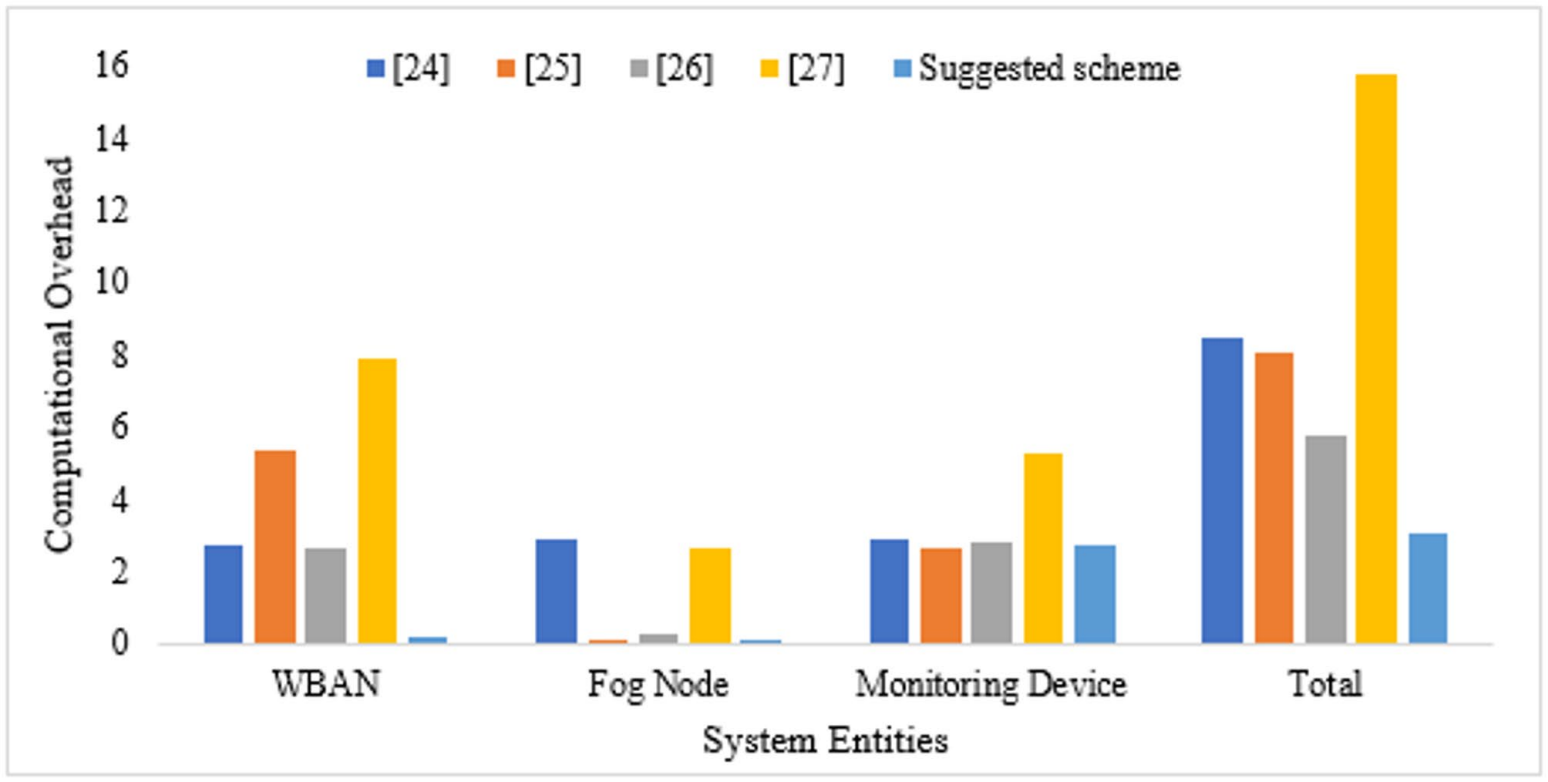
Computational overhead of various methods.

Figure 2 compares the computational overhead of the proposed approach with those of other existing competitive schemes^23–26^ and ensures the efficiency of the proposed approach.

#### Communication overhead

We analyze the total number of messages exchanged as well as the size of the data transmitted in the session establishment phase of the proposed authentication scheme to evaluate the communication overhead. Calculating all parts of the transmitted messages, including identifiers, nonces, hashed values, and challenge-response pairs. The scheme consists of four message exchanges among system entities during authentication and session key establishment. The total size of the first message (M1) sent from the WBAN Controller to a Fog Node is 64 bytes including a unique identifier16 bytes, a nonce of 16 bytes, and a hashed value of 32 bytes. The second message (M2) that the Monitoring Device sends to the Fog Node contains a Unique Identifier of 16 bytes, a nonce of 16 bytes, and a hash of 32 bytes, for a total of 64 bytes. The third message (M3) sent from the Fog Node to the WBAN Controller is a challenge-response pair of 16 bytes, a hashed value of 32 bytes, and a total size of 48 bytes. The Fog Node also sends a message (M4) to the Monitoring Device with 48 bytes, which contains a challenge-response pair of 16 bytes and a hashed value of 32 bytes, for a total of 48 bytes.

The total communication overhead of the proposed scheme is the sum of the following message sizes: 64 + 64 + 48 + 48 = 224 bytes. In a resource-constrained WBAN environment, this efficient design reduces data transmission while keeping robust authentication and session key establishment process^28^. Table 3 lists the communication overhead of various methods.

**Table 3:** Communication overhead of various schemes.

Methods	No. of messages	Overhead (bytes)
^24^	4	356
^25^	5	360
^26^	6	456
^27^	4	372
Suggested scheme	4	224

**Fig. 3:**
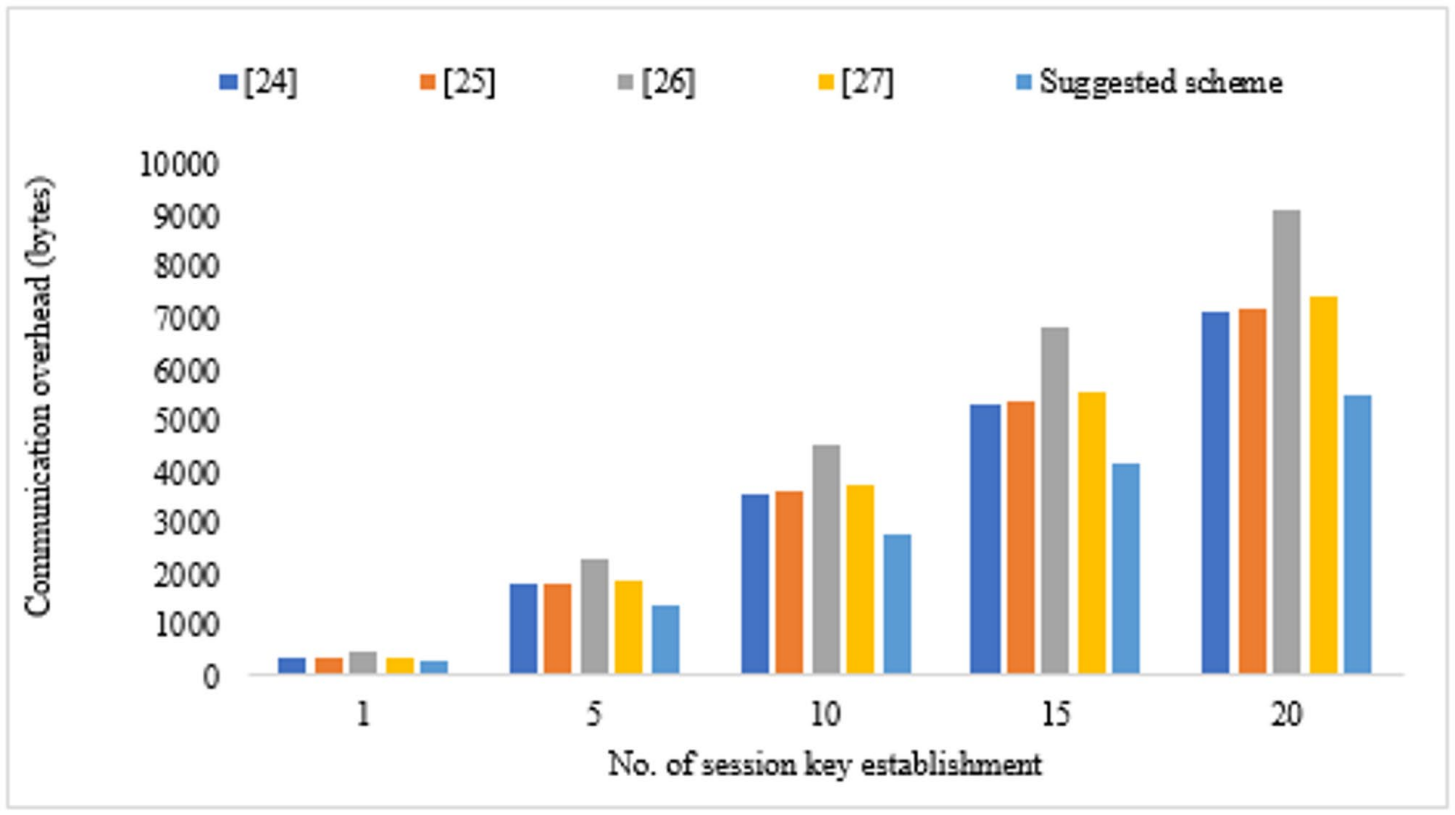
Communication overhead of various schemes.

A proposed scheme is shown to exhibit the lowest communication overhead among compared methods while preserving robust security features. This scheme provides a highly efficient communication protocol by reducing the size and number of transmitted parameters. This efficiency makes this protocol especially attractive for WBAN environments with resource constraints, where there is a need to minimize bandwidth and energy consumption. Figure 3 compares the communication overhead of the proposed scheme with other competitive schemes and shows that the proposed scheme outperformed other independent schemes in terms of reduced data transmission.

#### Security attributes

The security attributes of the proposed approach were evaluated and compared with other related schemes^23–26^ to ensure its performance efficiency. Table 4 compares the security attributes of the proposed approach and related schemes.

**Table 4:** Security attributes of various schemes.

Security attributes	^24^	^25^	^26^	^27^	Suggested scheme
Authentication	$\:Yes$	$\:Yes$	$\:Yes$	$\:No$	$\:Yes$
Unauthorized User Device Attack	$\:Yes$	$\:Yes$	$\:Yes$	$\:Yes$	$\:Yes$
Impersonation Attack	$\:No$	$\:No$	$\:No$	$\:No$	$\:Yes$
Replay Attack	$\:Yes$	$\:Yes$	$\:Yes$	$\:Yes$	$\:Yes$
User Anonymity	$\:Yes$	$\:Yes$	$\:Yes$	$\:Yes$	$\:Yes$
Forward Secrecy Attack	$\:Yes$	$\:Yes$	$\:Yes$	$\:Yes$	$\:Yes$
Temporary Secret Key Leakage Attack	$\:Yes$	$\:Yes$	$\:Yes$	$\:No$	$\:Yes$
Insider Attack	$\:No$	$\:Yes$	$\:No$	$\:Yes$	$\:Yes$
Physical Attack	$\:Yes$	$\:Yes$	$\:Yes$	$\:Yes$	$\:Yes$

The data in Table 4 demonstrate that the examined technique safeguards against unauthorised user device, replay, user anonymity, forward secrecy, and physical attacks. Conversely, schemes^23,25^ lack support for security aspects such as impersonation attacks and insider attacks. Similarly, the method in^24^ lacked protection against impersonation attacks. The^26^ scheme also lacks security features such as authentication, impersonation attacks, and temporary secret key leakage attacks. Conversely, the proposed strategy encompasses all the security attributes delineated in Table 4.

#### Storage overhead

The storage requirements for each entity in the proposed system are as follows: All registered devices need to have $\:CRPs$ stored in the $\:CS$. It takes 16 × 2 = 3216 × 2 = 32 bytes for $\:CRPs$ each device. It also has to store the master keys of other entities in the system: $\:{s}_{CS}$, $\:{s}_{{FN}_{i}}$ and $\:{s}_{{WBAN}_{j}}$​​​, for a total of 3 × 16 = 48 bytes3 × 16 = 48bytes. Additionally, it keeps track of the $\:UIDs$ of all devices, consuming 16 bytes per device. The total storage requirement for the $\:CS$ is 32 + 48 + 16 = 96 bytes.

For Fog Node, it must store the $\:CRPs$ 16 × 2 = 32 bytes, shared key 16 bytes, and $\:UIDs$ refrigerate joined contraptions as 16 bytes. The total fog node storage requirement is 64 bytes. The WBAN Controller must store the $\:CRPs$ 16 × 2 = 32 bytes, The WBAN specific key 16 bytes, and the Nonces and Temporary Identifiers 2 × 16 + 32 = 64 bytes. In total, the WBAN controller must store 32 + 16 + 64 = 112 bytes. The monitoring device must store the monitoring device key 16 bytes, Nonces, and Temporary Identifiers 2 × 16 + 32 = 64 bytes. In total, the monitoring device must store 16 + 64 + 16 = 96 bytes.

## Conclusion

In this paper, a physically secure and fog-capable lightweight authentication scheme for WBANs is presented. WBAN environments introduce specific challenges that the proposed framework addresses by combining the decentralised processing power of fog computing with the hardware-based security of PUFs. As shown by the proposed scheme, the reduction in the computational overhead is 64.33%, whereas the reduction in the communication overhead grid is 29.58% compared to the existing state-of-the-art WBAN devices. Results from the security analysis show that the proposed framework is robust against a variety of attack vectors, such as impersonation, replay, insider, and tampering. The scheme also accommodates key security properties, including mutual authentication, user anonymity, forward secrecy, and compromise resilience to temporary secret key leakages. Storage overhead analysis shows the context in which cryptographic parameters are stored by different actors, such that security is traded for resource efficiency. It provides a scalable, energy-efficient, and secure framework for WBANs using integration of lightweight cryptographic protocols, fog-enabled architecture, and PUF-based authentication. The results presented in this work provide a solid foundation for future research to further improve the scalability and adaptability of WBANs operating in a fog-enabled IoT ecosystem.

## Data Availability

The dataset underlying this study is publicly available on Figshare at 10.6084/m9.figshare.28540859.
